# Evaluation of the Effect of Mock Code Simulations on Code Blue Outcomes in a Saudi Tertiary Care Hospital

**DOI:** 10.7759/cureus.109988

**Published:** 2026-05-31

**Authors:** Mohammad Ismail, Waleed Kattan

**Affiliations:** 1 Department of Respiratory Therapy Administration, King Abdullah Medical City, Makkah, SAU; 2 Department of Health Services and Hospital Administration, Faculty of Economics and Administration, King Abdulaziz University, Jeddah, SAU

**Keywords:** acls adherence, code blue, in-hospital cardiac arrest, mock code simulation, tertiary care hospital

## Abstract

Background and aim: Mock code simulations have been employed to enhance resuscitation preparedness. However, support evidence from adult Saudi tertiary care hospitals remains scarce, particularly concerning their correlation with actual Code Blue outcomes rather than solely simulated performance. This study assesses whether implementing an in-situ mock code simulation program is associated with improvements in Code Blue outcomes and resuscitation performance metrics. This study aimed to provide context-specific evidence on changes in resuscitation process indicators and clinical outcomes, thereby supporting institutional efforts to strengthen emergency preparedness and patient safety in Saudi tertiary care practice.

Methods: A quantitative retrospective pre/post-intervention study was conducted utilizing Code Blue records from January to December 2025, including all adult patients with documented Code Blue incidents within the hospital, and analyzed 37 adult Code Blue records. The variables that were analyzed in this study included demographic characteristics, process indicators, advanced cardiovascular life support (ACLS) adherence metrics, and clinical outcomes.

Results: Primary clinical outcomes, return of spontaneous circulation (ROSC) (pre: 13/20, 65.0%; post: 13/17, 76.5%) and 28-day mortality, showed no statistically significant differences. Significant improvements were observed in ACLS adherence as follows: 2-min pulse time documentation increased from five (31.3%) to 16 (94.1%), rhythm identification increased from nine (56.3%) to 15 (88.2%), and epinephrine for non-shockable rhythms increased to 100.0% (17/17).

Conclusions: No statistically significant differences were observed in primary clinical outcomes, including return of spontaneous circulation and 28-day mortality. The most notable findings were ACLS adherence, particularly during each 2-min pulse check and 2-min rhythm check, and epinephrine administration in non-shockable rhythms. Mock code significantly enhanced ACLS adherence and quality process, although no immediate improvements were noted in survival outcomes. These results suggest that simulation-based training should be maintained and expanded as a strategy to strengthen resuscitation processes and team performance.

## Introduction

Cardiac arrest in hospitals is an emergency that is time sensitive, and survival relies greatly on the quality, responsiveness, and coordination of the hospital response [1-3]. This reality has elevated Code Blue performance to a patient safety and quality indicator, especially in tertiary care units where the complexity of cases, heterogeneity of diverse units, and variability in operations predispose delays and process disruptions [1-3]. The quality of resuscitation necessitates prompt arrest identification, instant initiation of basic life support, prompt defibrillation, and stipulated compliance with advanced cardiovascular life support (ACLS) algorithms. However, real-world practice is often limited by communication breakdowns, role misunderstandings, inconsistent documentation, and variations in location and shift. In most hospitals, despite available ACLS standards and formal Code Blue systems, avoidable performance gaps persist, including delayed cardiopulmonary resuscitation (CPR) initiation, delayed defibrillation in shockable rhythms, poor drug timing, inconsistent adherence to the algorithm, and poor team coordination [4]. The burden of in-hospital cardiac arrest (IHCA) is amplified in tertiary care hospitals, which manage patients with complex conditions and higher baseline acuity that may increase the risk of deterioration and complicate resuscitation. Furthermore, real exposure to Code Blue events is uneven across providers and units creating a readiness problem in which preparedness must be intentionally maintained through structured strategies, such as simulation, rather than relying on incidental experience alone [5].

This problem has led to the incorporation of simulation-based education, especially mock code and in-situ simulation, as a realistic approach to enhance technical performance and non-technical team behaviors during the resuscitation process [6,7]. In-situ simulation improves the transfer of training since it employs the same physical space, equipment, and workflow that clinicians would experience in real emergencies, while also disclosing unmanifested safety risks [6,7]. Previous studies tend to show improvements in key process indicators, such as faster CPR initiation, improved algorithm compliance, and improved team communication [8]. However, improvements in clinical outcomes, such as return of spontaneous circulation (ROSC) and survival, are less consistent and may depend on the program's intensity and duration, as well as the institutional setting [9].

In Saudi Arabia, there is current evidence of the viability and benefit of structured mock code training, primarily in the pediatric environment. In the meantime, there is a knowledge gap regarding evidence-based training in adult tertiary care. The extent to which mock code and in-situ simulation methods can translate into measurable adult patient outcomes in Saudi tertiary care facilities is not well reported [10,11]. Consequently, context-specific evidence is lacking regarding whether implementing a mock code simulation program has any positive effect on actual Code Blue outcomes in an adult Saudi tertiary care environment [10,11]. To address this gap, this study evaluates whether implementing an in-situ mock code simulation program at a Saudi tertiary care hospital was associated with measurable differences in adult Code Blue performance and outcomes. The intervention consisted of five mock simulation sessions, initiated on July 1, 2025, delivered across day and night shifts, with different specialties and workforce configurations. This study compares documented Code Blue events before and after implementation.

## Materials and methods

Study design and setting

This study used a retrospective quasi-experimental pre- and post-intervention design at a Saudi tertiary care hospital admitting adults with complex cases. The intervention was an in-situ mock code simulation program launched on July 1, 2025, comprising five sessions. Each session was conducted unannounced in actual care units, using standard clinical equipment, including crash carts and defibrillators, and followed the typical Code Blue workflow. Teams of physicians, nurses, and respiratory therapists participated according to standard shift and unit assignments, mirroring real Code Blue dynamics. Each simulation scenario concluded with a structured debriefing by a trained instructor, focusing on team performance, adherence to protocols, and improvement strategies. The design divided cases into the following two timeframes: before the intervention and after all five sessions.

Population and sampling

All adult patients who had a documented Code Blue incident at a Saudi tertiary care hospital during the study period from January 2025 to December 2025 comprised the study population. This study employed a non-probability total enumeration method, also known as census sampling, by including all eligible records. Exclusion criteria included patients who had not received full medical or event records, pregnant women, and patients with do-not-resuscitate (DNR) or do-not-intubate (DNI) status. The total population was 41 cases of Code Blue.

Data collection and variables

Data were collected from monthly cardiopulmonary resuscitation (CPR) committee reports at a Saudi tertiary care hospital using a standardized form. The main outcomes were clinical and process/performance outcomes. Clinical outcomes included return of spontaneous circulation (ROSC), patient outcomes, and 28-day mortality after cardiac arrest. Process outcomes included team leader arrival, time to start CPR, CPR length, time to first epinephrine, number of doses, time to first shock, and time to intubation. Several variables also measured ACLS adherence.

Data analysis

Data analysis was conducted using the Statistical Package for the Social Sciences (SPSS) version 26 (Armonk, NY: IBM Corp.). For categorical variables, comparisons were performed using the chi-square test; when expected cell counts were small, Fisher's exact test was preferred. For continuous variables, the independent-samples t-test was used when the variable was approximately normally distributed, and the Mann-Whitney U test was used when it was non-normally distributed. A p-value less than 0.05 was deemed statistically significant.

## Results

Screening and derivation of the analytical sample

Based on the study design, the mock code simulation program was deemed to have started on July 1, 2025. Since July 2025 was considered the initiation period for the intervention, the cases in July 2025 were treated as a transition period in the main analysis. Analysis of the dates in the PRE dataset showed four Code Blue incidents in July 2025. This was not considered the major pre-intervention and post-intervention comparison to maintain a clearer analytical distinction between the baseline and post-implementation periods.

Following the elimination of the transition-period cases, the analytical sample was 37 adult Code Blue events. Among them, 20 cases were described during the pre-intervention period, and 17 during the post-intervention period. Table 1 presents the derivation of the final sample.

**Table 1:** Screening and derivation of the analytical sample.

Screening stages	n
Pre-file: non-blank extracted records	24
Post-file: non-blank extracted records	17
Total extracted non-blank records	41
July 2025 transition-period cases excluded from primary analysis	4
Final pre-intervention sample	20
Final post-intervention sample	17
Final analytical sample	37

As shown in Table 1, the initial extraction yielded 41 non-blank Code Blue records. Exclusion of the four July 2025 transition-period cases yielded a final primary analytical sample of 37 eligible records, comprising 20 pre-intervention and 17 post-intervention cases.

Baseline characteristics

Table 2 indicates that there was no statistically significant difference between the pre-intervention and post-intervention groups in age, body mass index (BMI), length of stay, sex distribution, shift of occurrence, or witnessed arrest status (all p>0.05). The mean age was slightly higher in the pre-intervention group (59.6 vs. 55.1 years), and both groups were predominantly male (70.0% and 76.5%). Both groups fell within a similar median range for BMI (26.4 vs. 25.3). According to these findings, the two groups were largely similar in key baseline characteristics before the results and process indicators were compared.

**Table 2:** Baseline characteristics of the pre-intervention and post-intervention groups.

Variables	Pre-intervention (n=20)	Post-intervention (n=17)	p-Value
Age (years), mean±SD	59.6±14.6	55.1±12.7	0.324
BMI, median (IQR)	26.4 (23.0-31.2)	25.3 (21.6-27.5)	0.385
Length of stay (days), median (IQR)	12.5 (3.8-31.2)	17.0 (8.0-30.0)	0.583
Male sex, n (%)	14 (70.0)	13 (76.5)	0.725
Night shift, n (%)	8 (40.0)	9 (52.9)	0.431
Witnessed arrest, n (%)	9 (45.0)	8 (47.1)	0.90

Clinical outcomes

Table 3 indicates that ROSC occurred in 65.0% of cases (13/20) during the pre-intervention period and 76.5% (13/17) during the post-intervention period. The same numerical pattern was observed in patient survival, with 65.0% of pre-intervention cases and 76.5% of post-intervention cases alive after the event. Deaths that occurred within 28 days of cardiopulmonary arrest were reported to be 40.0% (8/20) in the cases before the intervention and 52.9% (9/17) after the intervention. All these differences were non-significant (p>0.05).

**Table 3:** Comparison of clinical outcomes between the pre-intervention and post-intervention groups. ROSC: return of spontaneous circulation

Clinical outcome	Pre-intervention (n=20)	Post-intervention (n=17)	Test statistic (χ^2^)	p-Value
ROSC achieved, n (%)	13 (65.0)	13 (76.5)	0.77 (df=1)	0.495
Alive after event, n (%)	13 (65.0)	13 (76.5)	0.77 (df=1)	0.495
28-day mortality, n (%)	8 (40.0)	9 (52.9)	0.79 (df=1)	0.517

Process indicators

Table 4 indicates that the pre- and post-intervention groups did not differ significantly in time to team leader arrival, time to initiate CPR, CPR duration, time to first administration of epinephrine, or epinephrine dose (all p>0.05). The difference in time to first defibrillation was statistically significant (p=0.024). This was, however, found to apply only to a small number of shockable-rhythm cases and should therefore be taken with caution.

**Table 4:** Comparison of process indicators between the pre-intervention and post-intervention groups. *Based only on applicable shockable-rhythm cases.

Process indicator	Pre-intervention	Post-intervention	p-Value
Time to team leader arrival (min), median (IQR)	0 (0-1.0)	0 (0-1.0)	0.633
Time to initiate CPR (min), median (IQR)	0 (0-0)	0 (0-0)	0.303
CPR duration (min), median (IQR)	22.5 (16.7-31.2)	19.0 (16.0-31.0)	0.891
Time to first epinephrine (min), median (IQR)	4.0 (4.0-6.0)	4.0 (4.0-4.0)	0.165
Time to first defibrillation (min), median (IQR)*	6.0 (4.0-6.5)	11.0 (10.0-13.5)	0.024
Time to intubation (min), median (IQR)	5.0 (3.8-7.0)	6.0 (6.0-8.0)	0.066
Number of epinephrine doses, median (IQR)	3.0 (2.0-4.3)	3.5 (2.0-5.0)	0.637

ACLS adherence indicators

As shown in Table 5, three ACLS adherence indicators differed significantly between the two study periods. Documentation of every 2-min pulse time increased from 30.0% in the pre-intervention group to 94.1% in the post-intervention group (p<0.001) with χ^2^ of 14.01 and effect size of 0.65. Documentation of every 2-min rhythm identification increased from 55.0% to 88.2% (p=0.028) with χ^2^ of 4.35 and effect size of 0.36. In addition, documentation of epinephrine administration for a non-shockable rhythm, according to the American Heart Association (AHA) guidance, increased from 56.3% of applicable pre-intervention cases to 100.0% of applicable post-intervention cases (p=0.003), with a χ^2^ of 9.24 and an effect size of 0.53.

**Table 5:** Comparison of ACLS adherence indicators between the pre-intervention and post-intervention groups. *Percentages were calculated using applicable cases only. AHA: American Heart Association; ACLS: advanced cardiovascular life support; PEA: pulseless electrical activity; VT: ventricular tachycardia; VF: ventricular fibrillation; BLS: basic life support

ACLS adherence indicator	Pre-intervention (met/applicable), n (%)	Post-intervention (met/applicable), n (%)	p-Value
BLS initiated in <1 min	20/20 (100.0)	16/17 (94.1)	0.459
Epinephrine administered within 5 min	14/20 (70.0)	15/17 (88.2)	0.246
Appropriate rhythm-specific action (PEA/asystole management or shock if VT/VF)*	9/13 (69.2)	7/7 (100.0)	0.249
Every 2-min pulse time documented	6/20 (30.0)	16/17 (94.1)	<0.001
Every 2-min rhythm identification documented	11/20 (55.0)	15/17 (88.2)	0.028
Every 2-min pulse checked	14/20 (70.0)	16/17 (94.1)	0.097
Appropriate joules selected in shockable rhythm*	9/13 (69.2)	5/7 (71.4)	1.0
Epinephrine documented for non-shockable rhythm according to AHA*	9/16 (56.3)	17/17 (100.0)	0.003
Epinephrine documented for shockable rhythm according to AHA*	2/3 (66.7)	6/7 (85.7)	1.0
Amiodarone dose according to AHA*	3/5 (60.0)	6/6 (100.0)	0.182
Amiodarone timing according to AHA*	3/5 (60.0)	5/6 (83.3)	0.545

Table 6 demonstrates that rhythm-specific variables were based on varying numbers of applicable cases, particularly for shockable-rhythm and amiodarone-related measures. Accordingly, the applicable case counts are important for interpreting these findings, as small denominators may limit the statistical stability of percentage-based comparisons.

**Table 6:** Comparison of process indicators between the pre-intervention and post-intervention groups. AHA: American Heart Association; PEA: pulseless electrical activity; VT: ventricular tachycardia; VF: ventricular fibrillation

Rhythm-specific indicator	Pre-intervention applicable cases, n	Pre-intervention met, n (%)	Post-intervention applicable cases, n	Post-intervention met, n (%)
Appropriate rhythm-specific action (PEA/asystole management or shock if VT/VF)	13	9 (69.2)	7	7 (100.0)
Appropriate joules selected in shockable rhythm	13	9 (69.2)	7	5 (71.4)
Epinephrine documented for non-shockable rhythm according to AHA	16	9 (56.3)	17	17 (100.0)
Epinephrine documented for shockable rhythm according to AHA	3	2 (66.7)	7	6 (85.7)
Amiodarone dose according to AHA	5	3 (60.0)	6	6 (100.0)
Amiodarone timing according to AHA	5	3 (60.0)	6	5 (83.3)

Additional analyses

The sensitivity analysis revealed that omitting July did not significantly alter the overall trends in the results. The data completeness for most variables used in the main analysis was high. The data for all 37 cases were valid for the key demographic, contextual, process, and outcome variables; one key variable, the number of epinephrine doses, was missing for one case.

## Discussion

The pre-intervention and post-intervention groups did not differ significantly on key baseline variables, including age, sex, body mass index, length of stay, shift of occurrence, and witnessed arrest status. This comparison suggests that the observed differences between the two study periods are less likely to be due to an imbalance in the measured background profile of the cases, rather than to the period surrounding the intervention. Regarding the key clinical outcomes, ROSC numerically rose during the post-intervention period, though the increase was not significant. This study found no statistical difference in 28-day mortality between the pre-intervention and post-intervention groups. These results indicate that there was no statistically significant increase in the harder clinical outcomes investigated in the current sample of the mock code simulation program's implementation. The analytical sample was small, limiting statistical power, especially for distal outcomes such as mortality. As vital as the first phase of resuscitation is, long-term outcomes also depend on the severity of the underlying illness, the cause of arrest, the physiological reserve, and the quality of post-resuscitation care. This trend indicates that the current data conform to a well-known pattern whereby simulation effects may be easier to identify in proximal process measures than in distal patient outcomes [12,13].

In general, statistically significant improvements in most of these process indicators were not observed in the post-intervention period. The recorded median times to team leader arrival and to initiate CPR in both groups were considerably short, which may reflect the institution's already rapid baseline response in these areas. In a small sample, the range of performance enhancement that can be measured may be constrained when performance is already high. The only process indicator that indicated a statistically significant difference between study periods was time to first defibrillation. This finding should, however, be taken with caution, as it was derived from a very small number of applicable shockable-rhythm cases.

Statistically significant results in the current study were observed in adherence to ACLS. According to the American Heart Association (AHA), statistically significant improvements were found in all 2-min pulse time records, all 2-min rhythm identification records, and epinephrine records for non-shockable rhythms. These results suggest that the mock code simulation program had a more direct link to improvements in recorded quality and procedural discipline during resuscitation [14]. The statistical significance of the increase in documentation of the 2-min pulse time is also vital, as it indicates enhanced homogeneity in the procedures used to regulate the resuscitation cycle. Since one pill at the right time and timing-specific treatment routes are fundamental elements of ACLS practice, enhancing this indicator reinforces the view that simulation can strengthen both algorithm modification and implementation discipline during actual Code Blue events. Process and adherence measures are usually more susceptible to simulation-based interventions than hard clinical outcomes [14]. The reason is that mock code programs are primarily designed to improve the delivery of resuscitation by enhancing timing, sequencing, documentation, communication, and strict compliance with protocol [14-16].

Among the contextual findings, there was a difference in the distribution of Code Blue events across hospital locations between the pre-intervention and post-intervention periods. Location can be an important factor in a tertiary care hospital, affecting staff availability, the team's familiarity with work in the emergency department, monitoring, and intensive care, as well as quick access to equipment and support staff [17]. Sensitivity analysis revealed that omitting July did not significantly change the overall trends in the results [17,18].

Scientifically, its primary value is that it demonstrates a stronger association between the intervention and the chosen ACLS adherence and process-quality outcomes than with the more difficult clinical outcomes of ROSC and 28-day mortality. The results imply that simulation of codes as mock codes can enhance discipline and standardization of resuscitation practice during real-life Code Blue events, specifically in terms of ACLS compliance and documentation quality [18-21]. In policy terms, the study recommends that mock code simulation be recognized as a component of a more comprehensive approach to quality improvement and emergency preparedness rather than an isolated educational intervention [14,19-24].

## Conclusions

This study evaluated the hypothesis that Code Blue outcomes and resuscitation performance indicators would significantly improve the implementation of an in-situ mock code simulation program at an adult Saudi tertiary care hospital. Significant post-intervention improvements were observed in selected documentation and protocol-based indicators, specifically in 2-min pulse time documentation, 2-min rhythm identification documentation, and epinephrine documentation for non-shockable rhythms in accordance with AHA guidelines. These findings indicate that the association between the mock code simulation program and improvements in the quality, structure, and standardization of resuscitation practice was more pronounced than for the more challenging patient-centered outcomes examined in this dataset. Therefore, mock code simulation is considered a valuable quality-improvement and preparedness strategy in adult tertiary care environments, particularly for reinforcing ACLS compliance and resuscitation discipline during actual Code Blue events. Future research should include larger sample sizes, multicenter settings across adult tertiary care facilities, and extended follow-up periods to identify larger differences in clinical outcomes, including ROSC and mortality. Additionally, greater emphasis on documentation discipline during real-life Code Blue events is recommended, as improved reporting of primary ACLS interventions may enhance procedural consistency and audit accuracy.
